# THE LANDSCAPE OF ALZHEIMER’S DISEASE AND RELATED DEMENTIAS CAREGIVER COMMUNICATION RESEARCH

**DOI:** 10.1093/geroni/igac059.1477

**Published:** 2022-12-20

**Authors:** Debra Dobbs, Jessica Yauk, Harleah Buck

## Abstract

Alzheimer’s Disease and Related Dementias (ADRD) affect one in nine adults aged 65 and over. Past research has demonstrated the need for services that improve quality of care for those with ADRD and their caregivers. Those with ADRD are at a greater risk for negative health outcomes. Therefore, it is crucial that those with ADRD receive care consistent with their preferences. However, more research is needed to shed light on effective tools available and patterns of advance care planning (ACP) when it comes to those with ADRD and their caregivers. This symposium highlights the landscape of ACP communication among those with ADRD and their caregivers. The session begins with a study that explored experiences of apathy and preferences for dyadic-communication via mobile health (mHealth) among adults with mild cognitive impairment and their care-partners. The second presentation demonstrates the feasibility and effectiveness of a palliative care education in assisted living program for nurses and administrators on increasing ACP discussions with family caregivers of residents with dementia. The third presentation investigates ACP among African Americans with ADRD and their caregivers and the critical role the community can play in facilitating communication. The final presentation explores how ACP differs by race, ethnicity and ADRD status using data from the 2018 Wave of the Health and Retirement Study. Future directions for implementation of these innovations for improving ACP communication among caregivers and persons with ADRD will be discussed.

